# Lignin, tannic acid and gallic acid reduce sulfolane miscibility in water with implications for pollutant migration and water treatment

**DOI:** 10.1007/s43832-026-00369-4

**Published:** 2026-02-27

**Authors:** Erica Pensini, Renato Souza Lima Sant’Anna, Alejandro G. Marangoni

**Affiliations:** 1 Department of Civil, Environmental and Water Resources Engineering, College of Engineering, University of Guelph, 50 Stone Road East, Guelph, ON N1G 2W1 Canada; 2 Biophysics Interdepartmental Group (BIG), University of Guelph, 50 Stone Road East, Guelph, ON N1G 2W1 Canada; 3 Food Science Department, University of Guelph, 50 Stone Road East, Guelph, ON N1G 2W1 Canada

**Keywords:** Complex fluids, Mixing behaviour, Molecular interactions

## Abstract

Sulfolane is a groundwater and surface water pollutant. Phenolic and polyphenolic compounds are found in plants and are ubiquitous in natural environments. Sulfolane is fully miscible in pure water, but its interactions with polyphenols decrease its miscibility. Lignin, a polyphenol immiscible in water, phase separates sulfolane from water, potentially impacting its migration in water. Sulfolane also interacts with tannic acid (a polyphenol) and gallic acid (a phenol), as seen by Fourier transform infrared spectroscopy and mechanistic simulations. These compounds are highly water-soluble and do not induce the macroscopic separation of sulfolane from water, but they form smaller clusters with sulfolane. Polyvinyl pyrrolidone flocculates gallic acid-sulfolane clusters, promoting their separation into larger droplets. Also, ferric iron forms complexes with gallic acid and sulfolane, promoting their sorption onto zero valent iron particles to achieve a removal of 224 ± 18 mg sulfolane/g zero valent iron within 30 s.

**Graphical abstract:**

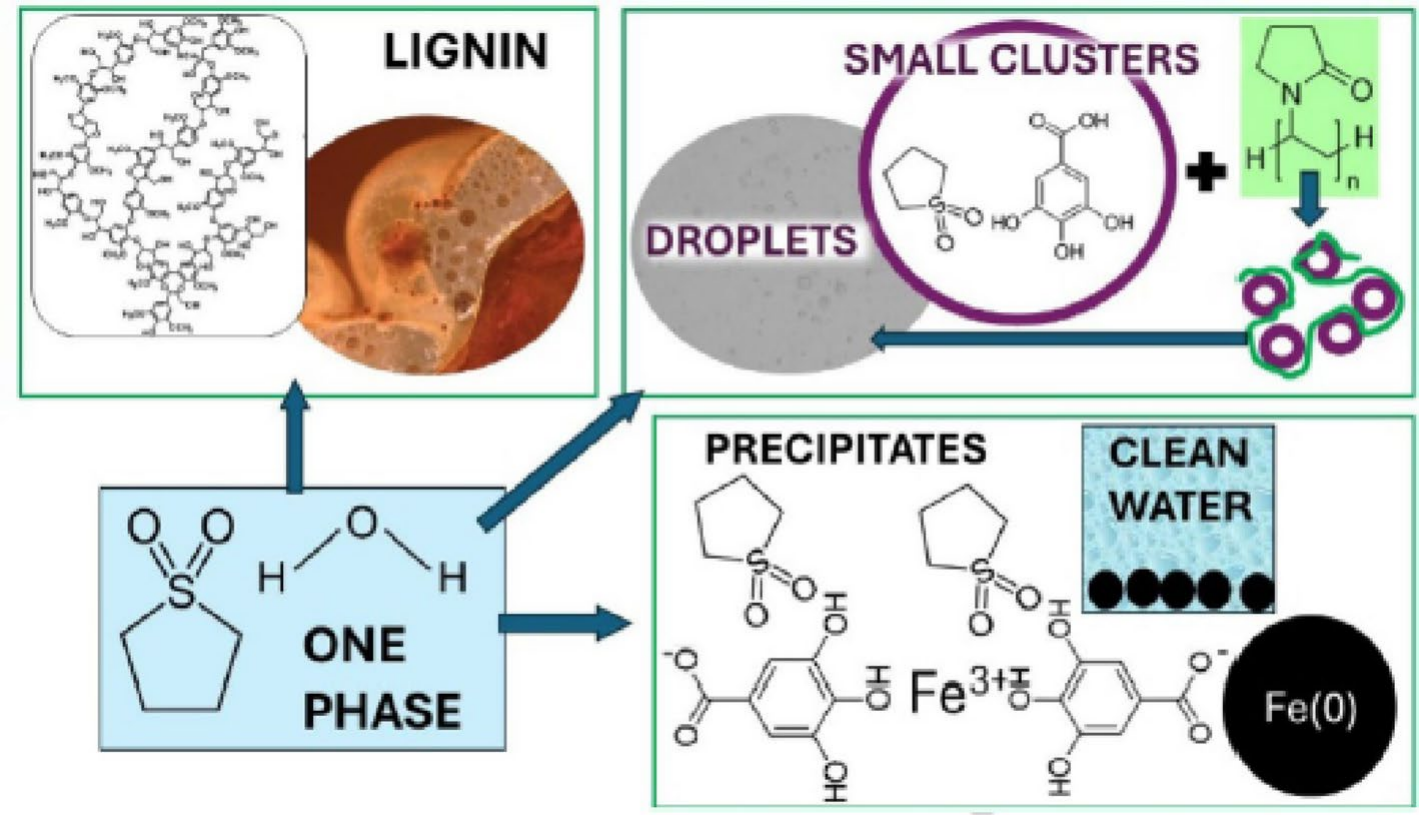

## Introduction

Sulfolane is a toxic organosulfur compound that is fully miscible in pure water [1, 2] and is utilized in multiple industrial processes [3]. For example, it is used to remove acidic compounds in natural gas processing and petroleum refining [4–6], and it also appears in solvent systems relevant to carbon capture and storage, commonly alongside amines [7–13]. Sulfolane contamination has been documented worldwide, including in Canada, Australia, the US, and Germany [14–17], typically linked to inadequate disposal practices or accidental releases [2, 18]. Environmental concerns extend beyond persistence in water, as sulfolane uptake by plants has been reported [19, 20], including food-relevant species such as leafy vegetables and fruiting plants [14] as well as garlic [21].

Although sulfolane is freely miscible in pure water, its aqueous phase behavior can be altered substantially by co-solutes and co-contaminants. Our previous work shows that salts—and sulfates in particular—decrease sulfolane miscibility [22, 23], thereby enhancing its sorption onto minerals such as clay [24]. Mechanistically, both sulfolane and sulfate accept hydrogen bonds from water, but sulfate–water interactions are stronger; sulfate therefore competes against sulfolane for hydration, destabilizing sulfolane’s molecular dispersion and promoting demixing. More generally, reduced miscibility can arise when nominally water-miscible pollutants preferentially associate with co-solutes to form clusters or aggregates with diminished effective hydration [25, 26].

Phenolic and polyphenolic compounds are particularly relevant in this context because they contain hydroxyl-rich motifs that can donate hydrogen bonds to strong acceptors. Directional hydrogen bonding and cooperative solvation between hydroxyl donors and strong acceptors are well-established in other oxygenate systems, including associations between sulfate-head surfactants and long-chain fatty alcohols [27, 28]. Toxic phenols (e.g., cresols, phenol, dimethylphenols) can occur as co-pollutants and have been reported to associate with sulfolane [29]. At the same time, benign phenolic structures are abundant in plants [30] and are pervasive constituents of natural organic matter in humus-rich soils, peatlands, and surface waters [31–34]. Whereas sulfolane miscibility is known to decrease in high-ionic-strength waters (e.g., sulfate-rich systems) [22–24], the extent to which phenolic/polyphenolic hydroxyl motifs can directly associate with sulfolane’s sulfonyl oxygens—and thereby alter sulfolane miscibility across molecular, mesoscopic (cluster), and macroscopic (phase separation) regimes—remains poorly constrained. Here, we address this gap by quantifying sulfolane mixing/phase behavior in the presence of representative phenolic/polyphenolic species and by resolving the underlying association mechanisms using Fourier transform infrared spectroscopy and mechanistic simulations.

We focus on lignin as an abundant, largely water-insoluble polyphenolic macromolecule and tannic acid as a water-soluble model polyphenol to isolate hydroxyl-rich motifs under aqueous conditions. We also investigate gallic acid as a small phenolic compound with potential utility for sulfolane removal from water. In addition to diagnosing association and phase behavior, we test two routes to convert dispersed sulfolane–phenolic associates into physically separable phases. First, we examine polymer-assisted separation in which polyvinylpyrrolidone (PVP)—a benign polymer known to bind polyphenols through hydrogen bonding [35–37]—acts as a bridging/flocculating agent that amplifies sulfolane–gallic-acid association into separable micron-scale droplets (e.g., by filtration or centrifugation). Second, we test coordination/precipitation-enabled capture: polyphenolic compounds such as tannic acid have been used to flocculate iron ions and phosphorus in water treatment [38], and to promote dewatering of oil sands tailings through polymer cross-linking and ferric coordination [39].

Building on these concepts, we use gallic acid to coordinate Fe(III), forming complexes that can bind sulfolane and foster its sorption onto magnetic zero-valent iron (ZVI) particles for rapid physical separation. We further include tert-butylhydroxyquinone (TBHQ) as a representative hydroxyquinone/phenolic additive that, in the presence of Fe(III), can form precipitated flocs enriched in sulfolane, consistent with prior reports of quinone–iron complex formation [40, 41].

Conventional sulfolane treatment approaches include oxidation, biodegradation by bacteria, and adsorption on activated carbon [2, 16, 42]. Activated carbon is often reported to outperform other sorbents (e.g., zeolites), which are more commonly deployed for other contaminant classes such as textile dyes or pharmaceuticals including paracetamol [43, 44]. The approach advanced here instead leverages phenolic-ligand-enabled association, coordination, and flocculation to drive rapid, physically separable capture (including seconds-scale kinetics and magnetic recovery) using comparatively benign reagents.

In this manuscript, we first quantify sulfolane mixing/phase behavior with lignin, tannic acid, and gallic acid. We then resolve sulfolane–phenolic association using Fourier transform infrared spectroscopy (FTIR) and mechanistic simulations. Finally, we demonstrate gallic-acid/Fe(III)-enabled capture on ZVI and polymer-assisted separation as rapid removal routes, and we position these findings relative to established remediation approaches, clarifying the practical niche and limitations for coordination-enabled capture and separable removal (Table 2).

## Materials and methods

### Materials

Sulfolane (99% pure), gallic acid monohydrate (≥ 98% pure), tannic acid (ACS reagent), lignin dealkaline (abbreviated as lignin, TCI), FeCl_3_∙6H_2_O (> 99% pure), iron (carbonyl iron powder, > 99.5% pure), polyvinylpyrrolidone PVP (MW 40,000), tert-butyl hydroxyquinone (TBHQ, TCI America) were purchased from Sigma Aldrich (Canada). Milli-Q water (18.2 MΩ·cm) was used in all experiments conducted.

### Attenuated total reflectance–Fourier transform infrared spectroscopy (ATR-FTIR)

ATR-FTIR was used for two purposes: (i) interaction screening/band assignment using neat (water-free) binary mixtures, and (ii) verification of sulfolane removal and phase enrichment in the FeCl_3_/zero-valent iron capture and TBHQ flocculation experiments, using the same aqueous formulations as in the corresponding bottle tests, as described below.

ATR-FTIR measurements were conducted on neat binary mixtures to screen molecular interaction interactions between phenolic compounds and sulfolane. Neat binary mixtures containing sulfolane and either lignin, tannic acid, or gallic acid were prepared at a 1:1 mass ratio (50:50 wt%). These measurements were intended to isolate sulfolane–polyphenol spectral signatures (–OH and carbonyl-region changes) without interference from bulk water.

ATR-FTIR measurements were also conducted on aqueous mixtures to verify sulfolane removal. Specifically, ATR-FTIR was performed on (a) the aqueous phase obtained after zero-valent iron particle separation/filtration in the sorption/capture tests (Sect.  2.6), and (b) the liquid phase and collected flocs (where present) from the TBHQ ± FeCl_3_ bottle tests (Sect.  2.4). These ATR-FTIR measurements were used to confirm sulfolane depletion in the aqueous phase and/or sulfolane enrichment in separated solids/flocs.

Instrument and acquisition parameters were the same in all cases. Absorbance spectra were collected using a Thermo Scientific Nicolet Summit FTIR with an Everest ATR accessory (IR Solution software). Each spectrum was averaged over 20 scans at 2 cm^−1^ resolution from 400 to 4000 cm^−1^. Experiments were conducted in triplicate (independent sample preparations) at minimum.

Spectral processing was also conducted consistently throughout. Spectra were processed using Quasar v. 1.5.0 (Orange Spectroscopy). Rubber-band baseline correction was applied. For qualitative comparisons, spectra were min–max normalized. For quantitative sulfolane determination (Sect.  2.6), the band-ratio approach described below was used without applying min–max normalization.

### Molecular modeling

Mechanistic simulations were performed to examine preferred sulfolane association motifs with gallic acid, tannic acid, and a model lignin structure in an implicit aqueous environment. Calculations were carried out using ChemSite Pro v. 10.5 and Molecular Modeling Pro Plus (MMP+) v. 8.1.40. AMBER force fields were used to minimize the potential energy of each system with water as the implicit solvent (dielectric 78.4; solute dielectric 1; GBV generalized Born model). The ChemSite Pro energy is reported as the sum of non-bonded, bonded (stretch/angle/torsion/improper), and implicit solvation terms. The simulation settings used were: timestep 1 fs; total time 10,000 ps; replay sampling period 200; equilibration steps 4000; nbi list refresh 20; cutoff distance 100 Å; implicit solvent water (solvent radius 1.4 Å); bath relaxation time 500 fs; with the “Non-bonded” and “Temp Gauge” options selected.

### Bottle tests

Bottle tests were used to qualitatively assess mixing behavior and visible phase separation/emulsion formation. Samples were prepared gravimetrically in glass vials, mixed by hand for 30 s, and then examined by optical microscopy (Sect.  2.5). The following formulation families were tested:

1. Sulfolane–water + polyphenols (phase behavior). Aqueous mixtures containing 10–30 wt% sulfolane relative to water were prepared, and polyphenols (lignin, tannic acid, or gallic acid) were added at 8–100 wt% relative to sulfolane (as applicable to each test set).
2. Polymer-assisted separation (gallic acid + PVP). Aqueous mixtures containing 30 wt% sulfolane relative to water were prepared with gallic acid at 1.5–15 wt% relative to the total mixture and polyvinylpyrrolidone (PVP) at 5 wt% relative to the total mixture. These samples were mixed by hand for 30 s and imaged by optical microscopy.
3. TBHQ flocculation screening (in the absence or presence of FeCl_3_). Mixtures containing 30 wt% sulfolane and TBHQ (0.06 M) were prepared in the presence and absence of FeCl_3_ (0.07 M). Samples were mixed by hand for 30 s, imaged by optical microscopy, and analyzed by ATR-FTIR (Sect.  2.2) to compare sulfolane enrichment in flocs versus the remaining liquid phase (where flocs formed).

### Optical microscopy

Samples prepared as described in Sect.  2.4 were imaged using a Keyence VHX-5000 digital microscope (Keyence Corporation, Canada) to document droplet/floc formation and macroscopic separation behavior. Representative images were collected for each formulation. Experiments were conducted in triplicate independent preparations.

### Sorption/capture tests (FeCl_3_–gallic acid–zero valent iron particles) and sulfolane quantification by ATR-FTIR

Sorption and water purification experiments were conducted using aqueous mixtures containing 30 wt% sulfolane relative to water, 2 wt% gallic acid relative to the total mixture, and FeCl_3_·6 H_2_O at the concentration used in the study (0.07 molal relative to total mixture mass and approximately 0.1 M relative to the water phase). Carbonyl iron powder (i.e., zero-valent iron particles, ZVI) was used as the sorbent.

After adding ZVI, samples were mixed by hand for 30 s. Iron particles were then separated by centrifugation for 1 min and the supernatant was filtered using 0.22 μm MCE filters (Fisherbrand; cat. 09-720-004) mounted on plastic syringes. The filtrate (aqueous phase) was analyzed by ATR-FTIR using the instrument settings described in Sect.  2.2.

Sulfolane concentration in the aqueous phase was quantified using a calibration curve based on the ratio of the water bending band δ(HOH) (1485–1814 cm^−1^) to the sulfolane sulfonyl stretching region (assigned as ν(S = O), 1056–1179 cm^−1^). Removal was calculated from initial and final aqueous sulfolane concentrations and reported as mg sulfolane removed per g ZVI (mean ± SD, *n* ≥  3).

## Results and discussion

### Overview of approach and formulation families

To connect molecular-scale association to macroscopic separation and capture, we combined four complementary elements: (i) ATR-FTIR on neat sulfolane–polyphenol binaries to identify diagnostic band shifts indicative of specific association motifs, (ii) implicit-solvent molecular modeling to probe preferred sulfolane–polyphenol arrangements and qualitative energetic trends, (iii) bottle tests with optical microscopy to assess aqueous phase behavior and droplet/floc formation, and (iv) capture tests in which sulfolane depletion from the aqueous phase was quantified by ATR-FTIR after isolating iron-containing solids. For clarity, the results are organized by technique, enabling direct comparison across polyphenol classes and making explicit how each dataset supports the proposed separation mechanism.

### Interactions between phenolic compounds and sulfolane, as probed by ATR-FTIR

ATR-FTIR was used to probe whether hydroxyl-rich phenolic/polyphenolic motifs interact with sulfolane in binary mixtures. Across lignin, gallic acid, and tannic acid, we observe consistent changes in the OH-stretch region upon addition of sulfolane, supporting hydrogen-bond-driven association between phenolic OH donors and sulfolane’s sulfonyl oxygens.

#### Lignin–sulfolane binaries

**Fig. 1:**
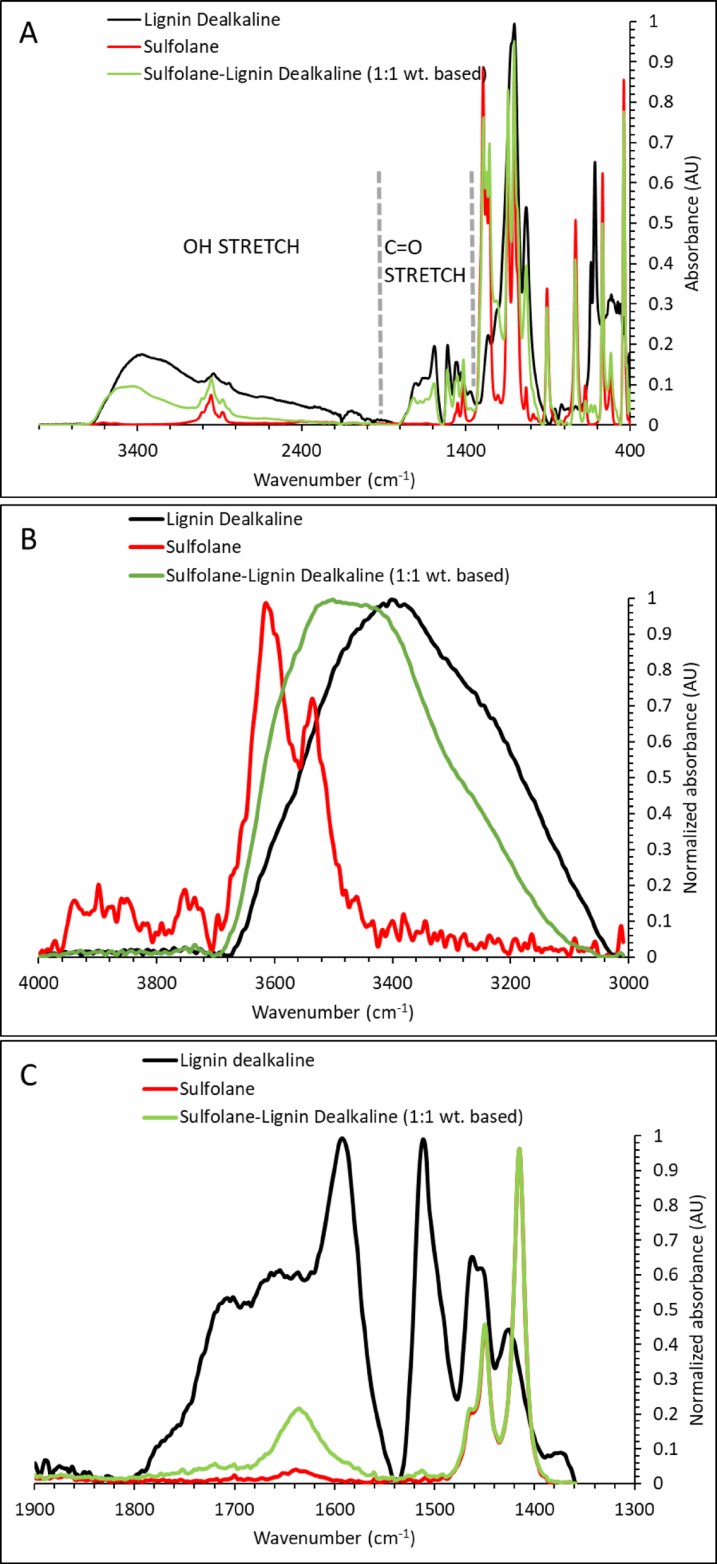
Absorbance spectra of lignin, sulfolane and their mixtures between 400 and 4000 cm^−1^ (**A**), with expanded views of the OH-stretch region (**B**) and the C=O region (**C**)

Lignin is insoluble in water at neutral pH, although basic pH promotes dissolution; lignin nevertheless has high affinity for sulfolane, which is used to prepare aqueous mixtures to extract lignin from biomass under basic conditions [45]. Here, ATR-FTIR reveals clear spectral changes upon mixing lignin with sulfolane (Fig. 1). The spectrum of neat lignin shows a broad OH-stretch band at ≈ 3000–4000 cm^−1^ [46]. Upon addition of sulfolane, this band blueshifts and becomes distorted (Fig. 1B), consistent with lignin OH groups engaging in new hydrogen-bond environments. Sulfate–hydroxyl interaction motifs have been noted previously [28], supporting the interpretation that lignin OH groups can act as donors to strong acceptors.

In the carbonyl region, neat lignin exhibits a band at ≈ 1550–1800 cm^−1^ that is commonly assigned to C=O-containing moieties [47–50]. This envelope comprises peaks at ≈ 1590, 1650, and 1700 cm^−1^ with a shoulder near 1768 cm^−1^ (Fig. 1C). Upon mixing with sulfolane, the ≈ 1650 cm^−1^ component redshifts to ≈ 1630 cm^−1^ and the relative intensity of the other components decreases. We interpret these changes as a redistribution of lignin’s hydrogen-bonding network: lignin OH groups interact preferentially with sulfolane’s sulfonyl group, which weakens (or reorganizes) intramolecular and intermolecular C=O···H–O interactions within lignin. Reported C=O frequency ranges for graphite oxide have been used to qualitatively relate carbonyl bands to hydrogen-bonding environments (e.g., terminal versus hydrogen-bonded C=O) [47–50]. Although lignin is chemically distinct from graphite oxide, such ranges provide a qualitative framework for interpreting how carbonyl environments can shift under changing hydrogen-bonding conditions.

#### Gallic acid– and tannic acid–sulfolane binaries

**Fig. 2:**
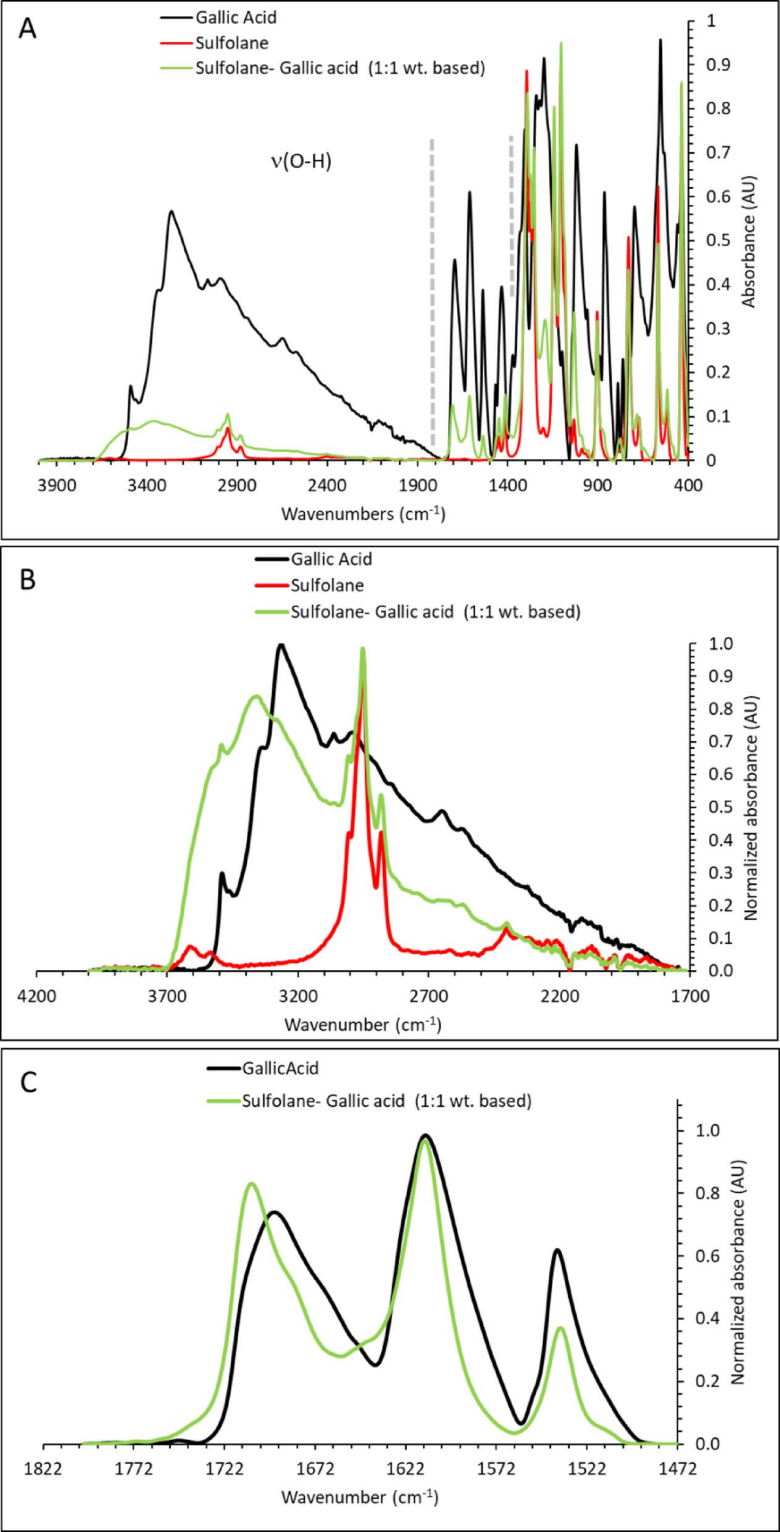
Absorbance spectra of gallic acid and its mixtures with sulfolane between 400 and 4000 cm^−1^ (**A**), with expanded views of the OH-stretch, ν(OH) (**B**) and C=O-stretch, ν(C=O) (**C**) regions

**Fig. 3:**
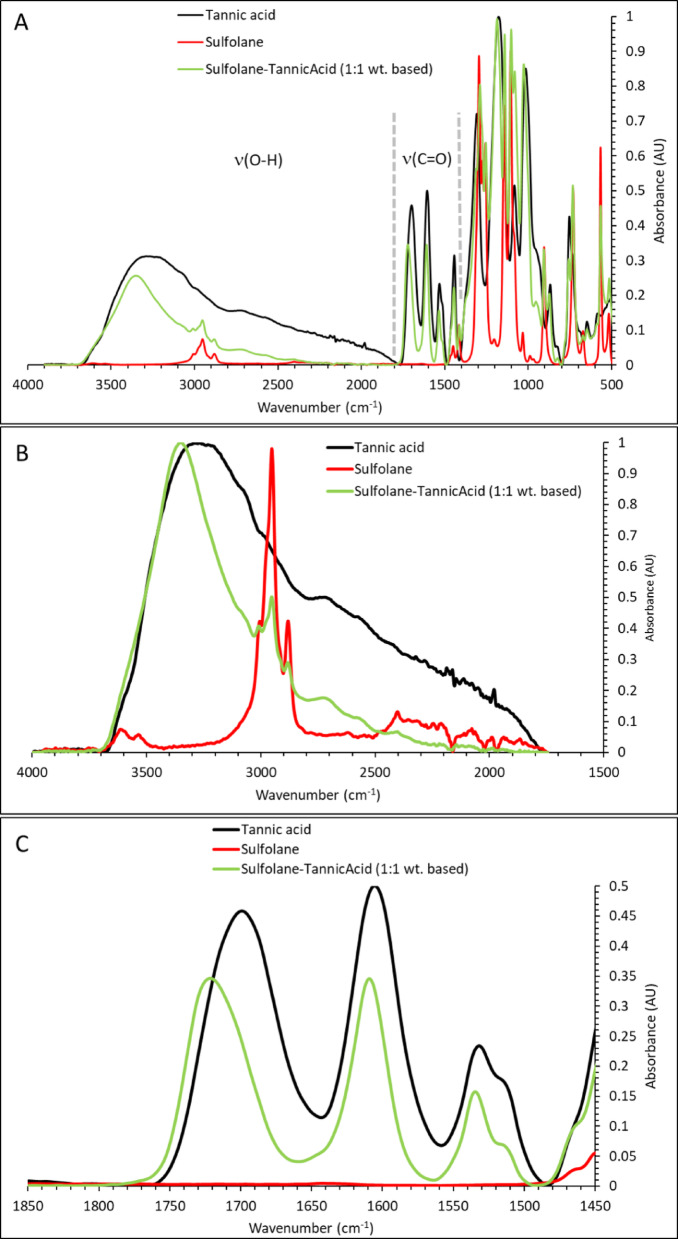
Absorbance spectra of tannic acid and its mixtures with sulfolane between 400 and 4000 cm^−1^ (**A**), with expanded views of the OH-stretch (**B**) and C=O-stretch (**C**) regions

We next examined the small phenolic compound gallic acid and the polyphenol tannic acid. In binary mixtures with sulfolane, gallic acid carboxyl groups are expected to be protonated. Both gallic acid and tannic acid display broad features in the ≈ 1700–3700 cm^−1^ region (Figs. 2 and 3), consistent with overlapping OH- and CH-stretch contributions; for tannin, OH-stretch peaks at 3280–3545 cm^−1^ and CH-stretch peaks at 2920–3200 cm^−1^ have been reported [51]. Upon mixing with sulfolane, gallic acid exhibits a blueshift of the OH-stretch band (Fig. 2B), consistent with the trend observed for lignin. For tannic acid, the OH-stretch envelope narrows and the lower-wavenumber components decrease (Fig. 3B), indicating a more uniform OH environment. We attribute this to preferential interaction of tannic-acid OH donors with sulfolane’s sulfonyl acceptors, reducing the diversity of OH···(OH/C=O/H_2_O) interactions present in neat tannic acid.

Consistent changes are also observed in the carbonyl region. Upon mixing with sulfolane, the C=O-stretch envelope of gallic acid and tannic acid in the ≈ 1500–1700 cm^−1^ region narrows, with diminished intensity of the lowest-wavenumber components (Figs. 2C and 3C). As for lignin, we interpret this as weakening (or reorganization) of C=O-centered hydrogen bonding upon introduction of sulfolane. Collectively, the OH- and C=O-region trends support the hypothesis that phenolic OH groups interact preferentially with sulfolane, rather than remaining engaged primarily in phenol–phenol (OH/C=O) or phenol–water hydrogen bonding.

### Interactions between phenolic compounds and sulfolane, as probed by mechanistic simulations

Mechanistic simulations were used to complement the ATR-FTIR results by assessing preferred sulfolane–phenolic arrangements and qualitative energetic trends in water (implicit-solvent representation). We simulated sulfolane with lignin, gallic acid, and tannic acid.

#### Lignin–sulfolane

**Fig. 4:**
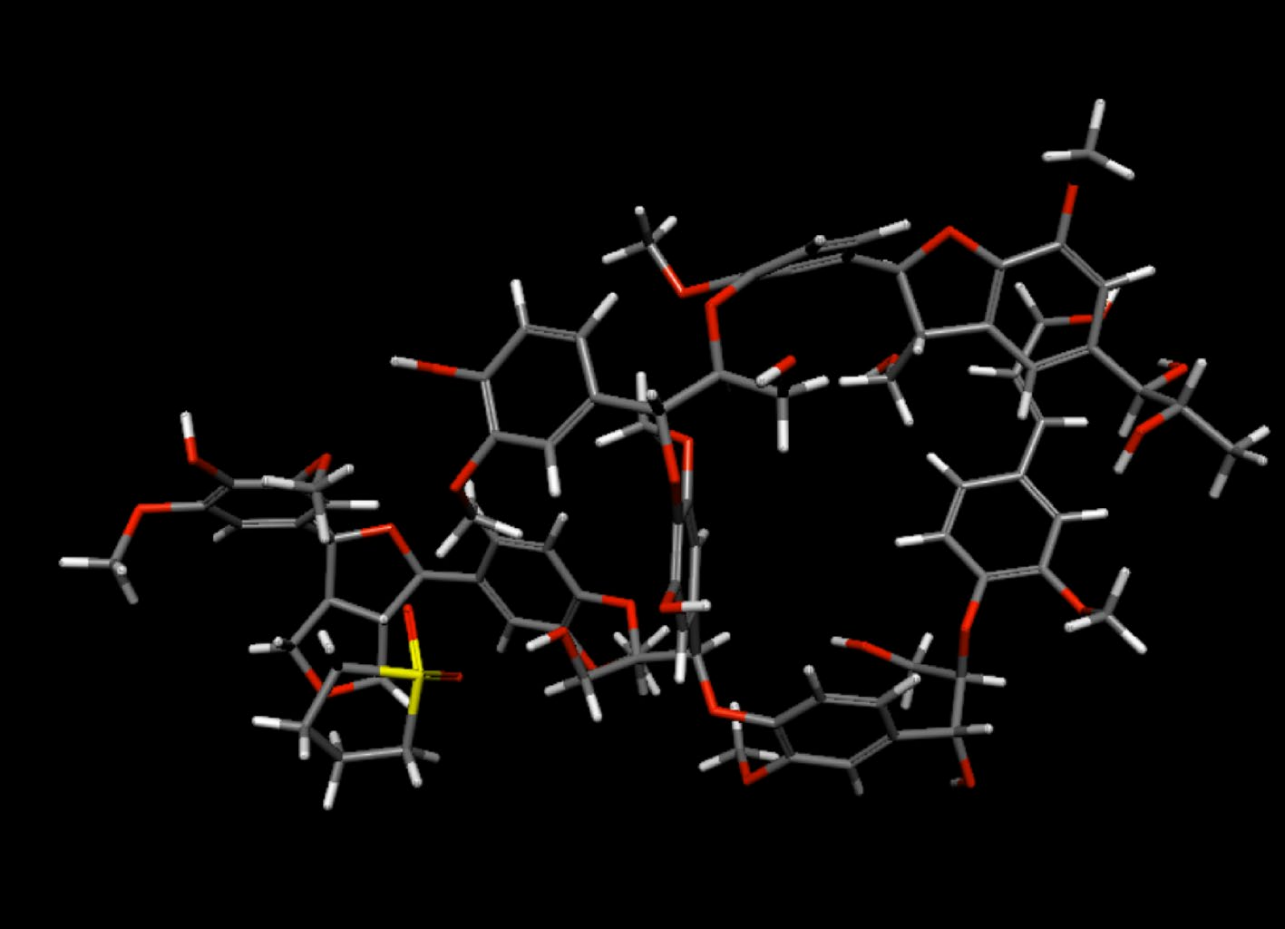
Optimized lignin–sulfolane structure for a system containing one model lignin and one sulfolane molecule, with water as an implicit solvent. Carbon atoms are grey, oxygen atoms are red, hydrogen atoms are white, and sulfur (in sulfolane) is yellow. The optimized geometry places lignin hydroxyl groups in close proximity to sulfolane sulfonyl oxygens, consistent with hydrogen-bond-driven association

The optimized lignin–sulfolane configuration (Fig. 4) places lignin hydroxyl groups proximal to sulfolane’s sulfonyl oxygens, consistent with the ATR-FTIR signatures discussed in Sect. 3.2. In the implicit-water model, the total energy of the combined lignin+sulfolane system is 14.55 kcal lower than the sum of the energies of the individually hydrated components. The non-bonded energy is likewise 15.2 kcal lower than the corresponding sum. We treat the non-bonded energy decrease as a qualitative indicator of favorable association between sulfolane and lignin within the chosen model chemistry. In this configuration, sulfolane acts as a hydrogen-bond acceptor, both with water and with lignin.

These observations align with our prior finding that water-miscible organics can be displaced from water by additives that engage water (e.g., by accepting hydrogen bonds) with comparable or greater strength, thereby restructuring hydration and favoring demixing [52]. On this basis, we propose that lignin can separate sulfolane from water because lignin–sulfolane association competes effectively with sulfolane hydration, consistent with both ATR-FTIR and simulations.

#### Gallic acid– and tannic acid–sulfolane

**Fig. 5:**
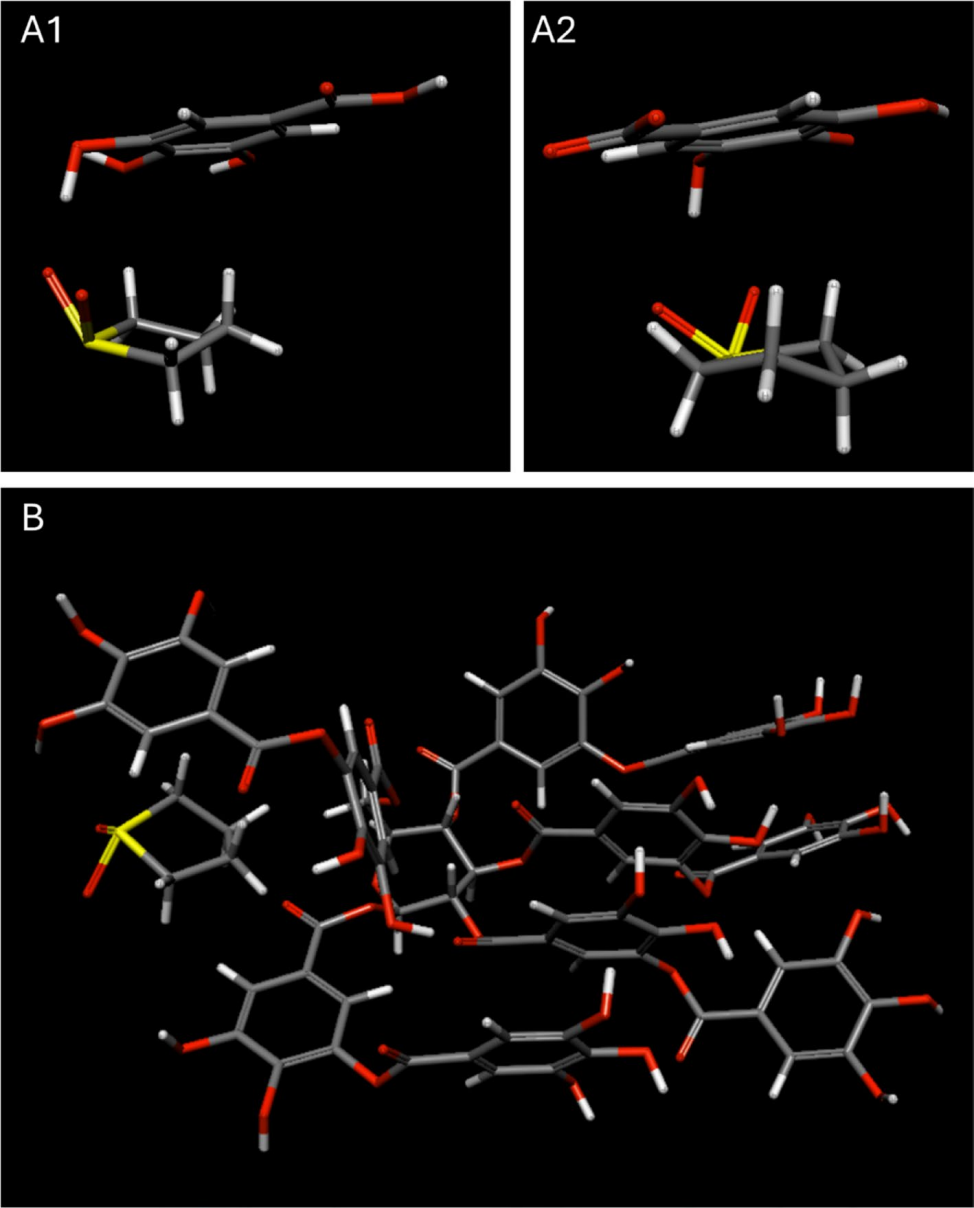
Optimized arrangements for sulfolane with (**A1**) gallic acid above the carboxyl pK_a_, (**A2**) gallic acid below the carboxyl pK_a_, and (**B**) tannic acid, with water as an implicit solvent. Carbon atoms are shown in grey, oxygen atoms in red, hydrogen atoms in white, and sulfur atoms (in sulfolane) in yellow

We next simulated sulfolane with gallic acid and tannic acid using the same implicit-water approach. Gallic acid can change protonation state depending on pH: the carboxyl group is protonated at acidic pH (pK_a_ ≈ 4.4) and deprotonated at neutral pH, while the phenolic OH groups have higher pK_a_ values (pK_a_ 2 ≈ 8, pK_a_ 3 ≈ 11, pK_a_ 4 ≈ 12). We therefore evaluated gallic acid–sulfolane association in two limiting regimes: (i) pH < pK_a_ of the carboxyl group (carboxyl protonated; all OH groups protonated) and (ii) 4.4 < pH < 8 (carboxyl deprotonated; OH groups protonated). The optimized configurations are shown in Fig. 5.

In both protonation regimes, simulations indicate favorable sulfolane–gallic-acid association (Table 1), with total energy differences of − 6.02 kcal (below pK_a_) and − 6.71 kcal (above pK_a_), and corresponding non-bonded energy differences of − 6.06 and − 6.03 kcal. Simulations likewise indicate favorable sulfolane–tannic-acid association (Fig. 5; Table 1), with a total energy difference of − 20.26 kcal and a non-bonded energy difference of − 18.31 kcal.

Although lignin, gallic acid, and tannic acid all show attractive association with sulfolane in this modeling framework, their effects on aqueous mixing behavior differ, as shown in Sects. "Mixing behaviour of sulfolane in water in the presence of lignin, as seen by bottle tests and optical microscopy"  and 3.5. This highlights that favorable molecular association is necessary but not sufficient to predict macroscopic demixing and motivates the separate phase-behavior analysis below.

**Table 1:** Energy differences between the sum of energies of individually hydrated components and the optimized paired system (sulfolane + phenolic compound)

System	Total energy difference (kcal)	Non bonded energy difference (kcal)
Gallic acid-sulfolane (below pKa)	− 6.02	− 6.06
Gallic acid-sulfolane (above pKa)	− 6.71	− -6.03
Tannic acid-sulfolane	− 20.26	− 18.31

### Mixing behaviour of sulfolane in water in the presence of lignin, as seen by bottle tests and optical microscopy

**Fig. 6:**
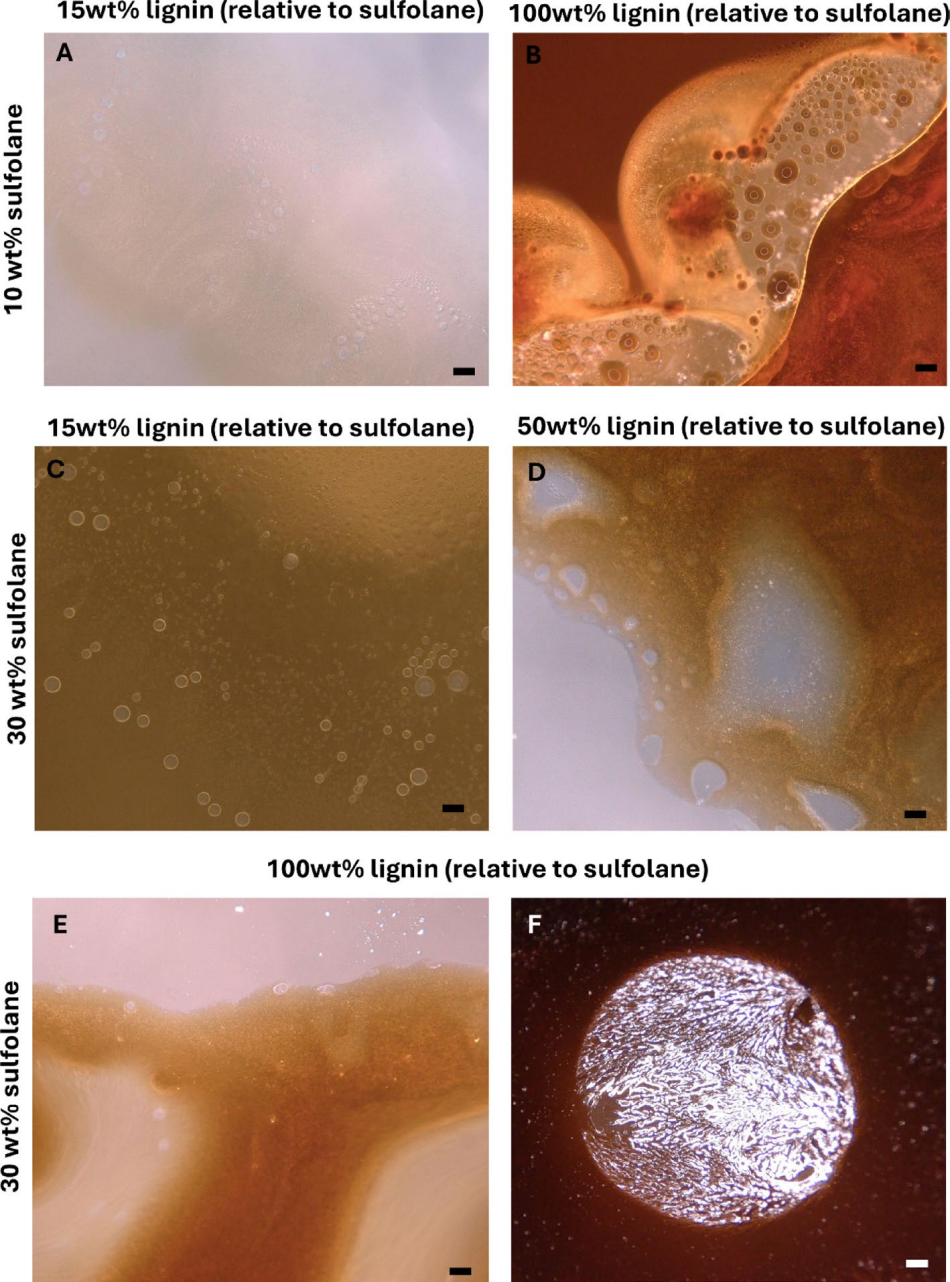
Optical microscopy images of mixtures containing varying weight % of sulfolane relative to water and varying weight % of lignin relative to sulfolane: 10 wt% sulfolane and either 15 wt% lignin (**A**) or 100 wt% lignin (**B**), 30 wt% sulfolane and either 15 wt% lignin (**C**) or 50 wt% lignin (**D**) or 100 wt% lignin (**E**, **F**). Scale bar: 50 µm

To test whether a polyphenolic macromolecule can demix sulfolane from water, we examined aqueous mixtures containing 10 wt% and 30 wt% sulfolane (relative to water) with varying lignin loadings (relative to sulfolane). Bottle tests and optical microscopy show that, while sulfolane and water are fully miscible in the absence of additives, lignin induces phase separation and the formation of macroemulsions (Fig. 6).

Microscopy reveals coexisting morphologies: water-rich domains containing dispersed sulfolane-rich droplets and, conversely, sulfolane-rich domains containing dispersed water droplets. Phase identity is qualitatively supported by color. Lignin is dark and sulfolane-soluble, imparting a deep-brown tint to sulfolane-rich phases, whereas lignin is largely insoluble in water, leaving the water-rich phase comparatively clear. Nonetheless, the water-rich phase retains some sulfolane and lignin. This is evident from crystalline residue that appears in otherwise transparent regions upon evaporation under the microscope (Fig. 6F). Collectively, these observations demonstrate that lignin can reduce sulfolane miscibility in water and promote sulfolane-rich domains, implying that phenolic-rich matrices could attenuate sulfolane mobility in natural environments.

#### Mechanistic interpretation

Integrating ATR-FTIR (Sect.  3.2) and simulations (Sect.  3.3), we propose that lignin-induced demixing reflects three coupled effects: (i) preferential hydrogen bonding between lignin OH donors and sulfolane sulfonyl oxygens (acceptors), (ii) competitive hydration, in which hydroxyl-rich motifs reduce the availability of water to stabilize sulfolane, and (iii) domain formation favored by lignin’s limited water solubility. In this framework, sulfolane is recruited into lignin-rich microdomains because it is an effective hydrogen-bond acceptor for hydroxyl-rich networks and can solvate less polar lignin motifs more favorably than water. Consistent with this picture, ATR-FTIR shows a blueshift/distortion of the lignin OH band upon sulfolane addition and redistribution of carbonyl-associated bands, indicating reorganization of lignin’s hydrogen-bonding environment. Simulations provide complementary support by indicating favorable lignin–sulfolane association within the chosen model chemistry.

#### Environmental significance

Sulfolane is often treated as fully miscible and predominantly molecularly dispersed in freshwater, with demixing primarily discussed for high-ionic-strength systems (e.g., sulfate-rich waters). The lignin results extend this framework by showing that hydroxyl-rich polyphenolic macromolecules can induce sulfolane-rich domains and macroemulsions even without salts as the primary driver. This suggests that in phenolic-rich environments (e.g., humic waters and peat-influenced systems), natural organic matter substructures may promote association and partitioning pathways that are not captured when sulfolane is modeled solely as a conservative, fully miscible solute.

### Mixing behavior of sulfolane in the presence of water-miscible phenolic compounds as seen by bottle tests and optical microscopy

Unlike lignin, which is largely insoluble in water at neutral pH, gallic acid is moderately soluble in water (≈ 1 g/L at 20 °C; solubility increases with temperature) [53], and tannic acid is highly water soluble (> 1000 mg/mL) [54]. We therefore examined whether these water-miscible phenolics can still measurably alter sulfolane mixing behavior.

#### Gallic acid and tannic acid without polymer

We prepared ternary mixtures of sulfolane, water, and either gallic acid or tannic acid, performed bottle tests to assess bulk-layer separation, and imaged samples by optical microscopy. Across the tested composition range (10–30 wt% sulfolane relative to water; up to 50 wt% gallic acid or 50 wt% tannic acid relative to sulfolane), no macroscopic phase separation or micron-scale droplets were observed by optical microscopy. The absence of optically resolvable droplets does not exclude molecular-scale association or submicron clustering. In our previous work, demixing can initiate via clusters too small to be detected optically, requiring scattering methods such as SAXS/SANS for direct observation [22].

These results highlight that macroscopic demixing depends on polyphenol solubility and effective valency. Lignin is both intrinsically water-insoluble at neutral pH and a multivalent polyphenolic network capable of forming a continuous phenolic-rich domain that can recruit sulfolane. In contrast, gallic acid and tannic acid remain sufficiently soluble that, while they can associate locally with sulfolane, the resulting associates remain dispersed at the concentrations examined and do not readily nucleate a separate phenolic-rich phase. This supports a continuum in which favorable molecular association can occur without macroscopic demixing when the phenolic component remains fully soluble, whereas demixing is favored when the phenolic component itself forms a distinct domain.

#### PVP-enabled droplet formation with gallic acid

**Fig. 7:**
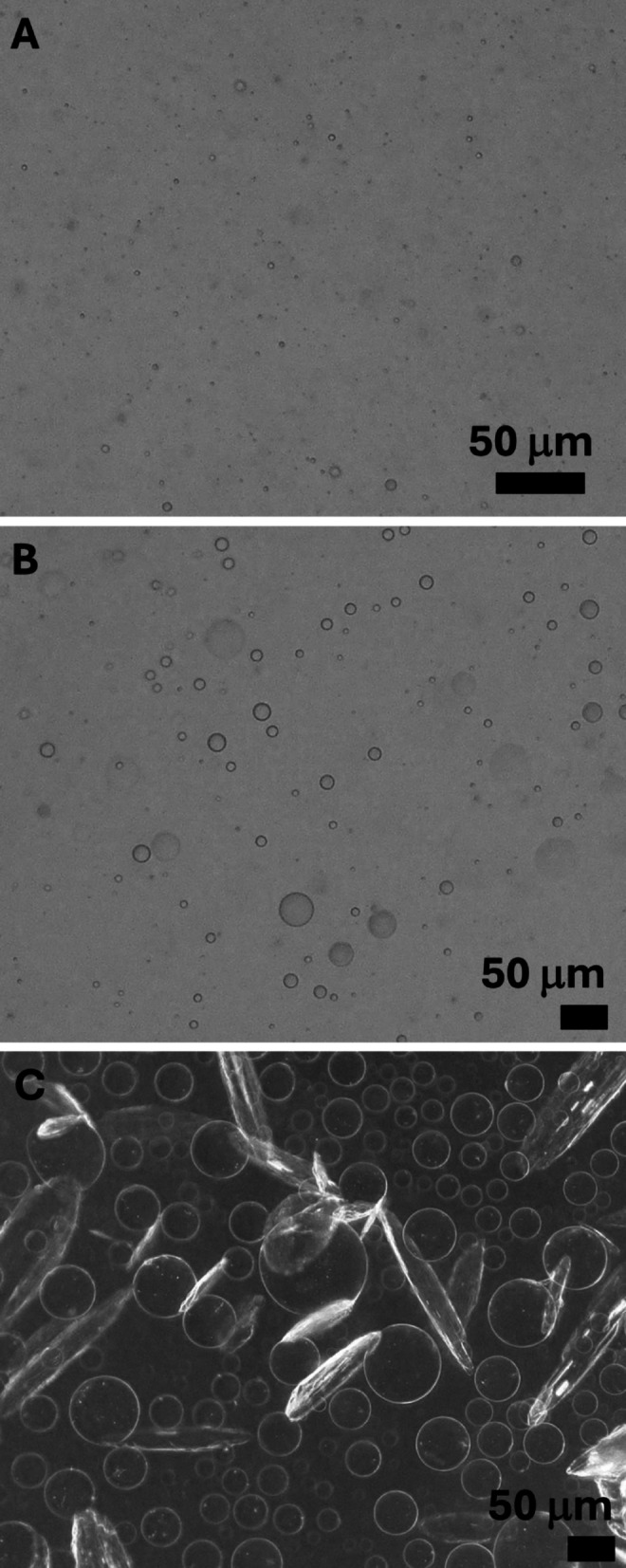
Optical microscopy images of sulfolane–water mixtures containing gallic acid and PVP: (**A**) 10 wt% sulfolane (relative to water), 5 wt% gallic acid and 5 wt% PVP (relative to total); (**B**) 15 wt% sulfolane (relative to water), 5 wt% gallic acid and 5 wt% PVP (relative to total); (**C**) 15 wt% sulfolane (relative to water), 15 wt% gallic acid and 5 wt% PVP (relative to total). Crystals appear at the highest gallic acid concentration (**C**), consistent with exceeding the saturation limit

Although gallic acid alone did not yield macroscopic separation, micron-scale emulsions were observed when polyvinylpyrrolidone (PVP) was added. Specifically, in mixtures containing 15 wt% sulfolane (relative to water) with either 4 wt% or 5 wt% gallic acid (relative to the total mixture) and 4.5 wt% or 5 wt% PVP (relative to the total mixture), optical microscopy revealed droplets (Fig. 7). By contrast, PVP alone (without gallic acid) did not induce sulfolane–water separation (data not shown). Droplets were also not observed at lower gallic acid levels (e.g., 1.5 wt% gallic acid with 5 wt% PVP and 30 wt% sulfolane), indicating an apparent concentration threshold for droplet formation under these conditions.

PVP was selected because it binds polyphenols in water, including gallic acid and tannic acid, through hydrogen bonding [35–37]. For example, Fan et al. report hydrogen bonding between the PVP carbonyl group and phenolic OH groups (e.g., tannic acid) [55]. We interpret the emergence of micron-scale droplets upon adding PVP as polymer-mediated bridging among gallic-acid–sulfolane associates/clusters: gallic acid acts as a molecular “recruiter” for sulfolane, while PVP provides multivalent binding sites that connect multiple polyphenol-bearing associates and promote aggregation into optically resolvable droplets/flocs. This polymer-assisted aggregation step converts dispersed associates into physically separable entities (e.g., by filtration or centrifugation).

Notably, droplets were not observed in 30 wt% sulfolane mixtures containing 50 wt% tannic acid (relative to sulfolane), even in the presence of PVP (5 wt% relative to the total mixture). This may reflect differences in phenolic architecture and/or the very high aqueous solubility of tannic acid [54], which can disfavor formation of a distinct phenolic-rich droplet phase under the tested conditions.

### Water purification approaches using benign phenolic compounds

 Because using low concentrations of any additive—even a benign one—is preferable, we explored a coordination-enabled capture route that uses gallic acid at low loading in combination with Fe(III) and zero-valent iron (ZVI) microparticles. Specifically, we tested sulfolane removal from mixtures containing 30 wt% sulfolane using 2 wt% gallic acid and 1.85 wt% FeCl_3_·6 H_2_O (relative to the total mixture), with ZVI particles (diameter ≈ 1–1.5 μm) as a magnetically recoverable solid phase. Polyphenols are known to coordinate ferric iron and promote flocculation [39], and Fe(III)–polyphenol complexation involves phenolic OH/phenolate groups [56, 57]; gallic acid in particular forms complexes with Fe^3+^ [58].

#### Observations and removal performance

 Adding gallic acid and FeCl_3_ to sulfolane–water mixtures in the absence of ZVI produced a dark solution with a green hue, with no optically resolvable droplets. When ZVI microparticles were added, the mixture cleared immediately after brief manual agitation (30 s) followed by magnetic separation or centrifugation/filtration. Under these short-contact conditions, sulfolane removal reached 224 ± 18 mg sulfolane per g ZVI within 30 s, emphasizing rapid kinetics and separability rather than equilibrium capacity optimization. Importantly, although FeCl_3_ lowers pH, acidification alone did not reproduce this effect: lowering pH with HCl to as low as ~ 1 did not lead to measurable sulfolane removal by ZVI in the absence of FeCl_3_.

#### Mechanistic interpretation

 The requirement for FeCl_3_ indicates that Fe(III) coordination chemistry—rather than pH reduction per se—is essential. We propose that Fe(III)–gallic complexes (formed through phenolic/phenolate groups) create sulfolane-enriched complexed species via hydrogen-bonding/solvation interactions, and that ZVI (and/or its nascent iron (oxyhydr)oxide surface) rapidly scavenges these species through adsorption/surface complexation and/or co-precipitation/bridging interactions, enabling efficient removal by magnetic separation and/or filtration. ZVI surfaces are positively charged near neutral pH [59] and can bind carboxylate-bearing species electrostatically, consistent with capture of Fe(III)–gallic-associated species.

#### Evidence for quinone-linked precipitation pathways and generality

 In the presence of ZVI, samples containing gallic acid progressively developed a deep purple hue, consistent with formation of oxidized phenolic species such as orthoquinones [59]. Quinones can form iron complexes [40], and single chelating o-semiquinone ligands and hydroxyquinones are known to complex Fe(III) [41]. In our experiments, deposits formed on glassware and were not readily removed by neutral water, consistent with limited solubility of these oxidized/complexed species. Limited solubility of Fe(III)–phenolic/quinone complexes provides a plausible basis for rapid, macroscopically apparent removal when ZVI is present as a high-surface-area sink that captures forming complexes/aggregates.

**Fig. 8:**
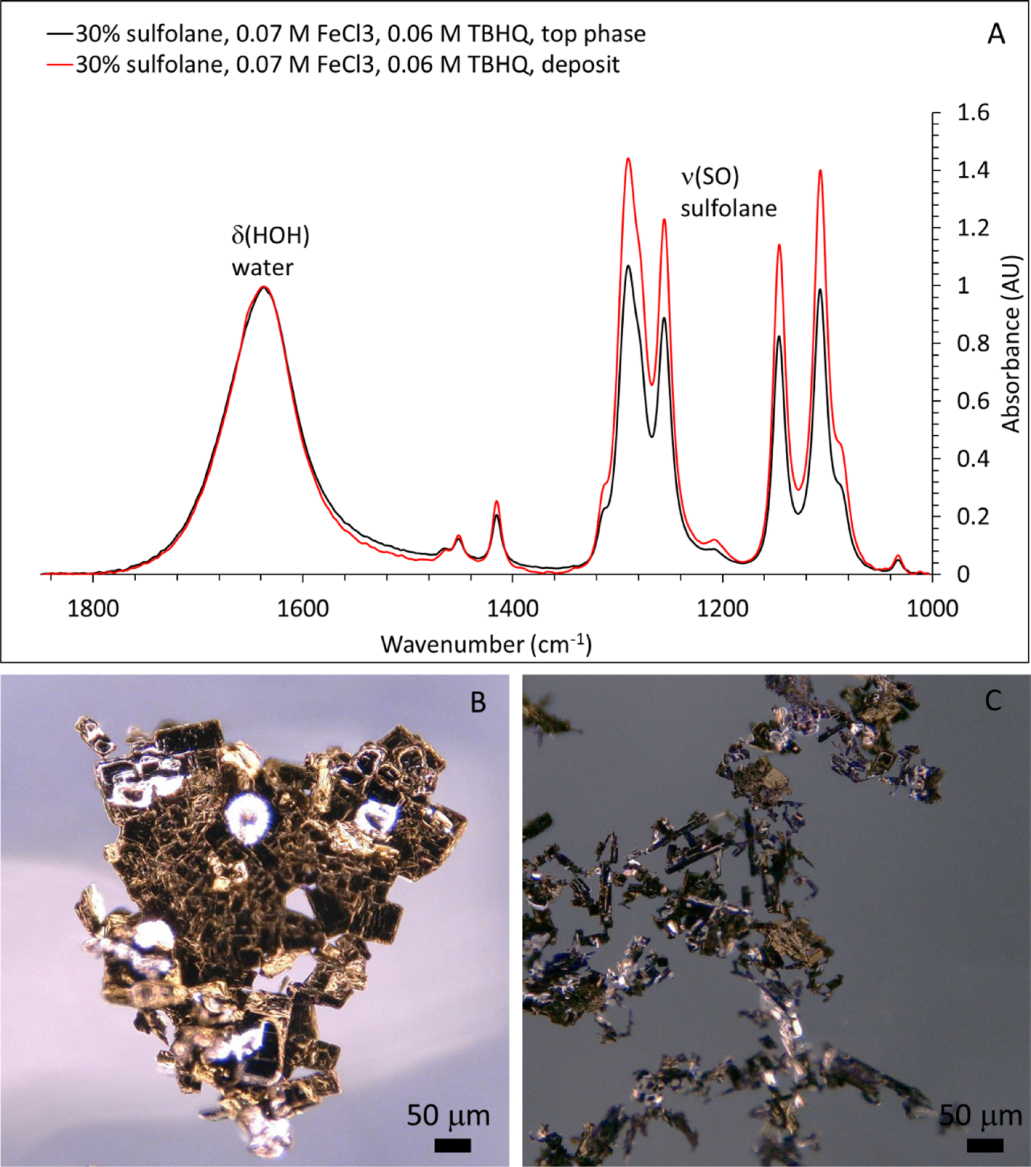
(**A**) ATR-FTIR absorbance spectra of the liquid phase and precipitated flocs formed in samples containing 30 wt% sulfolane in the presence of TBHQ and FeCl_3_. (**B**, **C**) Optical microscopy images of flocs formed with 0.07 M FeCl_3_ and 0.06 M TBHQ in aqueous solutions containing (**B**) 30 wt% and (**C**) 10 wt% sulfolane

To probe generality of coordination/precipitation-enabled capture, we conducted preliminary tests with tert-butylhydroxyquinone (TBHQ) and FeCl_3_ in 30 wt% sulfolane mixtures. In the presence of FeCl_3_, flocs formed and precipitated (Fig. 8); flocs were not observed without FeCl_3_. ATR-FTIR spectra show that these flocs are enriched in sulfolane relative to the aqueous phase (Fig. 8A), evidenced by a higher sulfolane-to-water band intensity ratio in the floc spectrum than in the liquid spectrum. Together, these results support a coordination-enabled capture concept in which Fe(III)–phenolic/quinone chemistry generates separable sulfolane-enriched phases that can be rapidly removed using magnetically recoverable iron solids.

### Comparison between the proposed approach and existing sulfolane remediation approaches

A range of remediation approaches has been proposed for sulfolane, including biodegradation, oxidation/advanced oxidation, and adsorption-based pump-and-treat. Under anoxic conditions, sulfolane attenuation is generally limited, whereas aerobic biodegradation is more promising [60]. Consistent with this, Kasanke et al. report biodegradation under aerobic conditions but not under nitrate-, sulfate-, or iron-reducing conditions [42]. Dinh et al. further note that oxidation methods such as advanced oxidation processes (AOP) and in situ chemical oxidation (ISCO) can be viable options requiring case-by-case assessment, and that pump-and-treat with granular activated carbon (GAC) is used at several sites [60]. Yu et al. similarly categorize oxidation and activated carbon as chemical and physical treatment approaches for sulfolane [16]. Adsorbent selection and water-matrix effects can strongly influence performance: activated carbon type matters [61], and natural organic matter (NOM) can occupy GAC sorption sites, limiting durability and effectiveness [61]. When appropriately designed, chemical oxidation and adsorption can act rapidly relative to bioremediation, which often depends on site redox state, microbial community acclimation, and contact times. For example, Izadifard et al. used advanced oxidation (activated persulfate with UV and UV–ozone) to treat sulfolane [62]. A summary comparison of these approaches is provided in Table 2.

The approach advanced here differs in both mechanism and operating envelope. Conventional strategies primarily emphasize transformation (oxidation/advanced oxidation; biodegradation) or non-specific capture (activated carbon and related sorbents). In contrast, we demonstrate a triggered, coordination-enabled capture pathway in which sulfolane is recruited into a ligand–metal/polyphenol association network and transferred onto a physically recoverable phase (magnetically separable iron particles). In parallel, we show that phenolic/polyphenolic motifs can measurably alter sulfolane’s apparent miscibility and promote aggregation, providing a mechanistic basis for sulfolane partitioning pathways that are not captured when sulfolane is treated as fully miscible under freshwater conditions.

Practically, the key distinction is not that coordination-enabled capture universally outperforms activated carbon or biological/oxidative treatments, but that it targets a different niche: rapid capture and separability under short contact times. In our proof-of-concept tests, Fe(III)–gallic-acid-assisted uptake onto zero-valent iron (ZVI) achieves seconds-scale kinetics and straightforward phase recovery by magnet/filtration, which may be advantageous for high-throughput or rapid-response treatment steps, or as a polishing step within a broader treatment train. The PVP-assisted aggregation results—while requiring higher additive loadings—support the same separation concept from an orthogonal direction: sulfolane–phenolic association can be amplified into separable micron-scale entities by introducing a second component that selectively binds polyphenols and promotes flocculation.

**Table 2:** Comparison of sulfolane remediation/separation approaches and the niche addressed in this study

Approach	Primary mechanism and objective	Typical contact time (qualitative)	Phase recovery/separation step	Matrix sensitivity (qualitative)	Key advantages	Key limitations/considerations
Oxidation	Chemical degradation (transform sulfolane)	Often minutes–hours (process-dependent)	Not applicable (contaminant transformed)	Often moderate–high (scavengers, alkalinity, NOM, etc.)	Mass reduction; mature engineering options	Energy/chemical demand; potential byproducts; strongly water-chemistry/process dependent
Biodegradation (natural attenuation or engineered bioremediation)	Microbial degradation (transform sulfolane)	Often days–weeks+ (site/community dependent)	Not applicable	Often high (electron acceptors, nutrients, acclimation, temperature)	Potentially low operating cost; in situ applicability	Slow/variable kinetics; uncertain reliability across sites; may require monitoring/conditioning
Activated carbon adsorption	Non-specific adsorption from water	Often minutes–hours to reach useful removal (system-dependent)	Filtration/carbon bed separation	Often moderate (competition with NOM, fouling)	Widely implemented; straightforward unit operation	Media replacement/regeneration; NOM competition; not inherently selective
Phenolic-enabled flocculation/separation (this study)	Triggered aggregation/phase transfer to create a separable sulfolane-enriched phase (association-driven demixing and/or coordination/bridging). Variants tested: (i) lignin-induced demixing/macroemulsions; (ii) gallic acid + PVP polymer-assisted aggregation; (iii) gallic acid + Fe(III) + ZVI capture onto recoverable solid (magnetic); (iv) quinone + Fe(III) precipitation/flocculation (preliminary).	Rapid once conditions met (seconds–minutes; 30 s proof-of-concept for Fe(III) + ZVI)	Physical separation (filtration/centrifugation/decanting; magnetic separation for ZVI variant)	High (pH, DOM/NOM/competing ligands; polymer–NOM interactions; Fe(III) speciation/ionic strength; lignin heterogeneity)	Distinct niche: short-contact-time capture + straightforward recovery; modular as a rapid step/polishing step; also establishes phenolic-driven partitioning pathway	Requires managing residual organics/iron and spent solids; performance likely varies with DOM/ligands; dosing/reproducibility depends on formulation; optimization needed for real waters

## Conclusions

Sulfolane is a pollutant found in groundwater and surface water, where phenolic and polyphenolic compounds are ubiquitous. While sulfolane is fully miscible in water, the polyphenolic compound lignin decreases its miscibility in water and causes it to phase separate, as seen by optical microscopy. This may impact the migration of sulfolane in groundwater, or else be used as the first step in treatment trains aimed at purifying water impacted by sulfolane. Sulfolane also interacts with the -OH groups of tannic acid and gallic acid, as observed by Fourier transform infrared spectroscopy and mechanistic simulations. These compounds alone do not separate sulfolane from water into large droplets, because of their higher solubility in water compared to lignin. Nonetheless, we propose that they form smaller clusters. Polyvinyl pyrrolidone (PVP), a polymer known to sequester phenolics from aqueous solutions, promotes the flocculation of sulfolane-gallic acid clusters, to form larger droplets visible by optical microscopy. Moreover, ferric iron forms complexes with gallic acid and sulfolane, promoting their sorption onto zero-valent iron (ZVI) particles to achieve a removal of 224 ± 18 mg sulfolane/g zero valent iron within 30 s. It is possible that ZVI particles reduce gallic acid to form quinones, which are less soluble in water. These form complexes with Fe(III) iron and sulfolane, and such complexes sorb more effectively onto ZVI particles compared to gallic acid-sulfolane complexes forming in the absence of Fe(III) ions. Sulfolane-enriched flocs also form in aqueous mixtures containing sulfolane, tert-butyl hydroxyquinone and FeCl_3_. In our future research, we will analyze the effect of different quinones on the separation of sulfolane from water, for the purpose of water purification.

The present study is a proof-of-concept and several practical constraints should be noted. First, coordination/flocculation pathways are expected to be sensitive to water chemistry (pH, ionic strength) and to competing ligands within dissolved organic matter that can sequester Fe(III) or otherwise disrupt the association network. Second, although the reagents employed are comparatively benign, any treatment implementation must consider residual ligands/polymers and the handling/disposal or regeneration of spent iron solids. Third, our microscopy-based observations do not resolve submicron clustering; future work using scattering methods and real-water matrices will be important to quantify cluster size distributions and to determine how these associations influence effective transport parameters under environmentally relevant concentrations.

## Data Availability

Data is provided within the manuscript and will be made available upon reasonable request to the corresponding author.
